# Supplementary material for: Kinetics and Thermodynamics of Adsorption for Aromatic Hydrocarbon Model Systems via a Coagulation Process with a Ferric Sulfate–Lime Softening System
Source: Materials (Basel). 2023 Jan 10;16(2):655. doi: 10.3390/ma16020655 (PMC9867294; doi:10.3390/ma16020655)
Supplement: Supplementary file 1 [file materials-16-00655-s001.zip › materials-2070943-supplementary.pdf]

Supplementary Materials

Kinetics and Thermodynamics of adsorption for Aromatic Hydrocarbon Model Systems via a Coagulation Process with a Ferric Sulfate–Lime Softening System

Deysi J. Venegas-García and Lee D. Wilson *

Department of Chemistry, University of Saskatchewan, 110 Science Place, Thorvaldson building, Saskatoon, SK S7N 5C9, Canada

* Correspondence: lee.wilson@usask.ca; Tel.: +1-306-966-2961; Fax.: +1-306-966-4730

Table S1: Selected analytical data† for South Saskatchewan River water used in this study.

HALOACETIC ACIDS		Drinking Water LIMIT*	Drinking Water	East Distribution	West Distribution
• Bromochloroacetic Acid	µg/L	none applied	1.53	1.59	1.59
• Dibromoacetic Acid	µg/L	none applied	<1.00	<1.00	<1.00
• Dichloroacetic Acid	µg/L	none applied	9.4	9.2	9.2
• Monobromoacetic Acid	µg/L	none applied	<1.00	<1.00	<1.00
• Monochloroacetic Acid	µg/L	none applied	<1.00	<1.00	<1.00
• Trichloroacetic Acid	µg/L	none applied	8.2	8.7	8.4
Halo Acetic Acids 5, Total (calc)	µg/L	80	17.7	17.9	17.5

TRIHALOMETHANES		Drinking Water LIMIT*	Drinking Water	East Distribution	West Distribution
• Bromodichloromethane	µg/L	none applied	5.1	5.9	5.6
• Bromoform	µg/L	none applied	<1.0	<1.0	<1.0
• Chloroform	µg/L	none applied	28.6	31.6	29.7
• Dibromochloromomethane	µg/L	none applied	<1.0	<1.0	<1.0
Total Trihalomethanes (calc)	µg/L	100	33.9	37.7	35.6

SPECIAL ORGANICS		Drinking Water LIMIT*	Drinking Water	Main Raw Water Intake
Benzene	mg/L	0.005	<0.00050	<0.00050
Benzo(a)pyrene	mg/L	0.00001	<0.0000050	<0.0000050
Carbon Tetrachloride	mg/L	0.005	<0.00050	<0.00050
1,2-Dichlorobenzene	mg/L	0.200	<0.00050	<0.00050
1,4-Dichlorobenzene	mg/L	0.005	<0.00050	<0.00050
1,2-Dichloroethane	mg/L	0.005	<0.0010	<0.0010
1,1-Dichloroethene	mg/L	0.014	<0.00050	<0.00050
Dichloromethane	mg/L	0.050	<0.0010	<0.0010
2,4-Dichlorophenol	mg/L	0.900	<0.00020	<0.00020
Ethylbenzene	mg/L	0.140	<0.00050	<0.00050
Monochlorobenzene	mg/L	0.080	<0.00050	<0.00050
Perfluorooctane Sulfonate (PFOS)	mg/L	0.0006	<0.000010	<0.000010
Perfluorooctanoic Acid (PFOA)	mg/L	0.0002	<0.000010	<0.000010
2,3,4,6-Tetrachlorophenol	mg/L	0.100	<0.00050	<0.00050
Toluene	mg/L	0.600	<0.00050	<0.00050
Trichloroethene	mg/L	0.050	<0.00050	<0.00050
2,4,6-Trichlorophenol	mg/L	0.005	<0.00030	<0.00030
Vinyl Chloride	mg/L	0.002	<0.00050	<0.00050
Xylene (Total)	mg/L	0.090	<0.00050	<0.00050

Table S1: continued

PESTICIDES and HERBICIDES		Drinking Water LIMIT*	Drinking Water	Main Raw Water Intake
Atrazine	mg/L	0.005	<0.00010	<0.00010
Bromoxynil	mg/L	0.005	<0.00010	<0.00010
Carbofuran	mg/L	0.09	<0.00010	<0.00010
Chlorpyrifos	mg/L	0.09	<0.00010	<0.00010
Dicamba	mg/L	0.12	<0.00010	<0.00010
Dichlorophenoxyacetic Acid (2,4-D)	mg/L	0.10	<0.00010	<0.00010
Diclofop-methyl	mg/L	0.009	<0.00010	<0.00010
Dimethoate	mg/L	0.02	<0.00010	<0.00010
Glyphosate	mg/L	0.28	<0.0050	<0.0050
Malathion	mg/L	0.19	<0.00010	<0.00010
MCPA	mg/L	0.10	<0.00010	<0.00010
Pentachlorophenol (PCP)	mg/L	0.06	<0.00050	<0.00050
Picloram	mg/L	0.19	<0.00010	<0.00010
Trifluralin	mg/L	0.045	<0.00010	<0.00010

†**Note**: The data above was authorized by Cleo Jahraus (Water Lab Coordinator) and Brian Sibley (Acting Plant Manager) in collaboration with the City of Saskatoon Water Treatment Plant and the Utilities and Environment Saskatoon Water Water Laboratory (December 2021).
